# Do descriptive norms messaging interventions backfire? Protocol for a systematic review of the boomerang effect

**DOI:** 10.1186/s13643-020-01533-0

**Published:** 2020-11-24

**Authors:** Jinyi Kuang, Maryann G. Delea, Erik Thulin, Cristina Bicchieri

**Affiliations:** 1 Center for Social Norms and Behavior Dynamics, University of Pennsylvania, McNeil Building, 3718 Locust Walk, Philadelphia, PA USA; 2 Gangarosa Department of Environmental Health & Hubert Department of Global Health, Rollins School of Public Health, Emory University, 1518 Clifton Road, Atlanta, GA USA; 3 RARE organization, 1310 N. Courthouse Road, Suite, Arlington, VA 110 USA

**Keywords:** Systematic review, Descriptive norms, Boomerang effect, Intervention, Social norms, Behavior change

## Abstract

**Background:**

Descriptive norms messaging interventions are used to motivate people to adopt or maintain desirable behaviors. Such interventions provide people with information that describes an undesirable behavior as uncommon or a desirable behavior as prevalent within a relevant social group. Descriptive norms messaging interventions have shown promise in increasing individual and social benefit for a broad range of health and sustainability programs. However, evidence suggests that people who have adopted desirable behaviors sometimes regress to undesirable behaviors after receiving descriptive norms messages due to the type of information provided in the messages. This phenomenon is called the boomerang effect. We aim to conduct a systematic review of boomerang effects on health and environmental sustainability behaviors resulting from exposure to descriptive norms messaging interventions.

**Methods:**

We will employ our search strategy to identify studies of descriptive norms messaging interventions published prior to December 31, 2020. We will search the Cochrane Library, Campbell Library, PsycINFO, PubMed, Social Science Research Network (SSRN), and Web of Science to retrieve peer-reviewed articles published in English. We will restrict inclusion to studies (e.g., randomized and non-randomized controlled trials, quasi-experimental studies, and observational studies) of health and environmental sustainability interventions that assess behaviors before and after exposure to descriptive norms messaging. Two reviewers will independently extract data about study populations and design, intervention components, and behavioral measures. We will use the revised Cochrane Risk of Bias assessment tool (RoB2) and Risk Of Bias in Non-randomized Studies—of Intervention (ROBINS-I) to assess the risk of bias, and the Liverpool Quality Assessment Tool (LQAT) to assess the quality of evidence. We will conduct thematic analyses to codify interventions, and examine intervention effects across subgroups of individuals based on their behavior prior to intervention exposure (e.g., those practicing desirable behaviors vs. undesirable behaviors). We will also conduct moderator analyses to determine whether boomerang effects are contingent upon other factors including intervention framing and delivery modality.

**Discussion:**

This systematic review will provide information about descriptive norms messaging intervention effects across subgroups of individuals and elucidate factors that potentially moderate boomerang effects. The review will yield evidence-based recommendations for the structure and content of descriptive norms messages that can be employed to avoid unintended boomerang effects within the context of health and sustainability programming.

**Systematic review registration:**

PROSPERO CRD42020156989

## Background

Humans are social beings; they seek information about what relevant others do and adjust their behavior to fit in to the group [1, 2]. When people believe that a crowd behaves in a certain way, they are likely to conform to what the crowd does [3]. These rules that govern behavioral expectations within groups are called social norms [4]. A behavioral pattern that is conditional only on a person’s belief about what is commonly done is known as a descriptive norm [5].

The power of conformity has inspired a type of behavior change technique called descriptive norms messaging. Interventions that employ descriptive norms messaging aim to motivate people to adopt or maintain desirable behaviors by providing people with information that describes an undesirable behavior as uncommon or a desirable behavior as prevalent within a relevant social group [3].

Program implementers and researchers have utilized descriptive norms messaging as an intervention technique to increase individual and social benefit for a broad range of public health and sustainability programming—from increasing vegetable consumption [6] and discouraging alcohol misuse amongst college students [7] to reducing plastic bag use [8]. However, evidence suggests that people who have adopted desirable behaviors sometimes regress to undesirable behaviors after receiving descriptive norms messages due to the type of information provided in the messaging [9, 10]. This phenomenon is called the boomerang effect [10]. The boomerang effect refers to when people who exhibit a desirable behavior become less likely to do so after being exposed to descriptive information about others that indicates the desirable behavior is less frequent, occurs to a lesser degree, or is not exhibited amongst other people in relevant social groups [10]. Studies examining boomerang effects after exposure to descriptive norms messaging interventions have yielded inconsistent results. One study, for example, found that households with relatively low energy use increased their energy consumption after receiving descriptive information about their neighbors’ average energy use, as the average was higher than their own relatively low consumption levels [10]. Conversely, a study of an intervention designed to correct college students’ exaggerated perceptions of alcohol use on college campuses found that lighter drinkers who drink less than the typical student did not increase drinking after receiving personalized descriptive norms messaging [11]. This inconclusive evidence regarding the intervention effects of descriptive norms interventions warrants further examination into potential, yet unintended boomerang effects that result from exposure to descriptive information regarding relevant others’ behaviors.

Previous systematic reviews have examined the effectiveness of descriptive norms messaging interventions on targeted behaviors amongst entire intervention populations [12–14] and the unintended boomerang effects of information-based interventions [15, 16]. However, to our knowledge, no known reviews have examined boomerang and differential effects of descriptive norms messaging interventions amongst subgroups of intervention populations based on their behavior prior to intervention exposure. To fill in this gap, our systematic review will synthesize evidence regarding potential boomerang effects of descriptive norms messaging interventions designed to change behaviors related to health and environmental sustainability. More specifically, the aim of this review is threefold. First, we aim to examine peer-reviewed literature evaluating descriptive norms messaging interventions that promote health and environmental sustainability behaviors. Second, we aim to synthesize evidence regarding intervention effects on subgroups that behave differently prior to intervention exposure, and determine the magnitude and direction of such effects on people who practice desirable behaviors prior to being exposed to descriptive information that indicates others practice the desirable behavior to a lesser extent or not at all (i.e., boomerang effect). Third, we aim to identify factors that account for heterogeneity of boomerang effects.

## Methods/design

This protocol has been registered within the PROSPERO database (registration number CRD42020156989) and is being reported in accordance with the reporting guidance provided in the Preferred Reporting Items for Systematic Reviews and Meta-Analyses Protocols (PRISMA-P) statement [17, 18] (see checklist in Additional file 1).

### Eligibility criteria

Eligibility criteria for this review align with the PICO-T framework, which defines populations, interventions, comparators, outcomes of interest, and types of studies eligible for review [19].

#### Types of studies

To allow for examinations of behavior pre- and post-intervention exposure, we will restrict our review to ex-ante randomized and non-randomized controlled trials, controlled before-and-after studies, interrupted-time-series studies, uncontrolled before-and-after studies, and case series (uncontrolled longitudinal studies).

#### Types of populations

We will review studies involving healthy populations, including children, adolescents, and adult population (regardless of age or sex).

#### Types of interventions

Interventions of interest for this systematic review include health and environmental sustainability interventions that employ descriptive norms messaging, where exposed groups receive summary information regarding others’ behaviors (e.g., proportion of others engaged in the desirable behavior, total number of people engaged in the behavior, mean or median number of people who have adopted the desirable behavior within a relevant social group). We will not limit inclusion based on the duration, frequency, or method of intervention exposure.

#### Types of comparators

We will include studies that make comparisons between groups receiving descriptive norms messaging interventions and counterfactual comparator groups (e.g., groups received alternative interventions or no interventions).

#### Types of outcome measures

The primary outcomes of interest are changes in behavior related to health and environmental sustainability after exposure to descriptive norms messaging interventions. Specifically, the measures of behavior change may include changes in the prevalence of the desirable behavior (e.g., proportion of people engaging in the behavior) or the degree or frequency of performance of the desirable behavior (e.g., the average degree or frequency of the behavior). As such, we will restrict studies to those that captured both pre-intervention and post-intervention measures of the desirable behaviors.

### Exclusion criteria

We will exclude studies if the description of study methods is incomplete or ambiguous to the extent that renders reviewers unable to determine whether the study meets all inclusion criteria. If authors fail to report sufficient statistics or data for estimating changes in behavior before and after intervention exposure, we will include the related studies in the review and thematic synthesis, but not the meta-analyses [20]. Exclusion criteria are subject to change during screening and selection. If changes are made, they will be reported in the final review paper and applied retroactively to all previously screened studies.

### Information sources and search strategy

We will harvest data from literature written in English and published in peer-reviewed journals prior to December 31, 2020. We will use search terms and Boolean operators to identify articles that may be eligible for review. We will systematically search the following electronic databases: Cochrane Library, Campbell Library, PsycINFO, PubMed, Social Science Research Network (SSRN), and Web of Science. For each database, we will first employ keyword searches (e.g., descriptive norms, health behaviors), then apply MeSH terms (if applicable), text words, and phrases associated with the keywords. We will then generate preliminary search results by combining all keyword search sets with appropriate Boolean operators. Literature generated from the searches will be exported into a reference management software (EndNote). Duplicates will be removed prior to screening. Our detailed search strategy for MEDLINE is described in Additional file 2. As various electronic databases have their own respective search platforms that operationalize search strategies, we will modify our search procedures, as needed based on the respective databases. We will report the full database search process, including all iterations used to search all targeted databases when our searches are complete.

### Study screening and selection

We will perform screening and selection in a stepwise manner. First, two reviewers will independently screen the titles and abstracts of papers identified during the database search, using our pre-established inclusion and exclusion criteria to identify eligible studies. The reviewers will exclude studies if their titles and abstracts (a) meet any exclusion criteria or (b) clearly fail to meet all inclusion criteria. If the reviewers are not able to make a definitive decision based on the title and abstract, they will independently review the full text of the paper to determine eligibility. The two reviewers will independently review the full text of all papers that are determined eligible upon title and abstract review such that studies included in the review meet all inclusion and no exclusion criteria.

If disagreements arise between reviewers during the study screening and selection process, they will be resolved by discussion with a third reviewer. If disagreements arise due to a lack of information, we will contact the primary study authors for clarification. We will record and report disagreements and their resolutions. We will use Cohen’s kappa to assess inter-rater reliability between reviewers [21].

### Data extraction and coding

Two reviewers will independently extract relevant data from all studies included in the review using a standardized data extraction form (see Additional file 3). We will pilot test the data extraction form using two randomly selected articles, and refine it, as needed. If relevant data are unclear or unreported, we will contact the authors for clarification. We will harvest data about study populations (including age and gender distributions) and settings (including country and region), details related to study design and interventions (including intervention details such as descriptive norms message design and any non-descriptive norms co-interventions, and control details, if any), and statistical methods. We will extract data on behavioral measures both pre- and post-intervention exposure. For dichotomous outcomes, we will extract the number of participants who practice desirable and undesirable behaviors and ratio measures with standard errors, if available. For count outcomes, we will extract the number of episodes the desirable or undesirable behaviors were practiced (e.g., frequency of excessive alcohol consumption per week across study arms with the total person-time in each study arm the rate ratio) and standard errors, if available.

We will extract statistical information from included studies based on the type of data collected, the type of statistical significance tests performed, the effect size measures used, and the types of statistics information reported. If the study specifically reports data related to individuals who outperform the descriptive norms messaging information in the pre-treatment measure, we will extract the pre- and post-treatment sample size. If the study reports the effect size *d*, it will be entered; if the study does not report effect size data for the groups of interest, but the authors report sufficient information, we will calculate the estimated effect size estimator *d*. If the study meets inclusion criteria but does not report sufficient data to calculate the effect size, we will contact the corresponding author to request the information or dataset, and perform effect size calculation ourselves. We will code effect sizes as positive if the performance of the behavior increased in the desired direction after intervention exposure. In contrast, we will code effect sizes as negative if the performance of the behavior does not increase in the desired direction after intervention exposure. If the authors indicate that the effect was simply “non-significant,” we will enter a zero effect size and *p* value of 0.50 [22, 23].

We are aware that the level of intervention and analysis will likely vary across studies. Some studies likely focused on individual behavioral change or maintenance while others likely addressed household-level behavioral change or maintenance. As a result, we will calculate and present effect estimates based on the level at which the descriptive norms interventions were administered (e.g., individual, household, community).

### Risk of bias assessments

Two reviewers will independently assess the risk of bias for each included study using the revised Cochrane Risk of Bias assessment tool (RoB2) for randomized trials and Risk Of Bias in Non-randomized Studies—of Intervention (ROBINS-I) for non-randomized studies of interventions [24, 25]. The assessments will be conducted at the study level, following the guidance accordingly. To eliminate the possibility of bias in assessing quality, author names and affiliations may be removed from reports before they are evaluated. We will report the assessment results and conduct a sensitivity analysis to estimate the potential impact of studies at high risk of bias on the overall conclusion.

### Quality of evidence appraisal

We will use the Liverpool Quality Assessment Tool (LQAT) to critically assess the quality of evidence included in the review [26]. We will use the following criteria to assess the quality of evidence presented in the review: (a) selection procedures, (b) baseline assessment, (c) outcome assessment, (d) analysis/confounding, and (e) contribution of evidence towards review questions that are rated as strong, moderate or weak.

### Data synthesis

We will conduct our analyses in two phases, which we summarize here and detail further in subsequent sub-sections. First, we will conduct qualitative analyses to codify intervention themes, examine descriptive norms messaging structure and content, and produce a narrative synthesis of studies included in the review. Then, we will examine overall trends in behaviors before and after intervention exposure, and the heterogeneity of results presented in included studies. We will carry out meta-analyses if it is appropriate to do so based on the heterogeneity of studies, targeted behaviors and levels of intervention, and behavioral measures. We will also assess publication bias and perform moderator analyses. We will perform our meta-analysis in RStudio (version 1.1.463) using the “metaphor” library to do so [27]. Our pre-defined type I error threshold is alpha = 0.05 on two-tailed tests.

#### Qualitative analyses

Given the scope of this systematic review and the descriptive nature of the intervention messages (i.e., providing statistical information of others’ behaviors), we anticipate heterogeneity amongst the included studies. Regardless of whether we are able to perform meta-analyses, we will tabulate the characteristics of the included studies and conduct a thematic analysis following the procedure suggested by Thomas and Harden [28]. During our qualitative analysis phase, we will code the intervention messages, organize them into descriptive themes, use those themes to investigate the types of intervention messages that are effective at changing behavior in the desirable direction, and identify factors that may moderate the results (e.g., targeted populations, behaviors of interest). We will report results by intervention domain (e.g., energy conservation, vegetable consumption, alcohol consumption) and separately for subgroups based on whether individuals practiced desirable behaviors prior to intervention exposure.

#### Meta-analyses

Given the scope of our research questions and the anticipated heterogeneity of studies emerging from various domains, we assume studies included in the review will not estimate the same underlying population parameters. Therefore, we will use random effects models and employ the restricted maximum likelihood estimation method [29]⁠.

To compute the weighted mean of the effect sizes, we will assign weight to certain studies based on the inverse of the associated fixed effects and/or random effects variances [30]. This method has been shown to outperform weighting by sample size in random effects analyses [31].

To test for heterogeneity of the effect sizes, we will use the homogeneity statistic (*Q*), which has a chi-square distribution with degrees of freedom equal to the total number of effect sizes minus one (*e* – 1) [27, 28]. We will also use the *I*^2^ statistic as a second measure of heterogeneity, which is more useful to compare across meta-analyses and is less dependent on the number of synthesized effects [30, 32]⁠.

Outlying effect sizes are defined as effect sizes that are three standard deviations larger or smaller than the mean. Following the winsorization method suggested by Dixon and Tukey, we will minimize any outlying effects and replace them with the next most extreme values [33, 34].

### Publication bias

We will address publication bias through the use of the following three strategies. First, we will address the “file drawer problem” by computing the number of fail-safe on individual effect sizes with Rosenberg’s calculator [35]⁠. Second, we will present funnel plots, with the *y*-axes representing study precision (i.e., error of the intervention effect estimates in a reverse scale) and the *x*-axes representing intervention effect size estimates (i.e., standardized mean difference), as suggested by Sterne and Egger [36]⁠. To statistically test and adjust for publication bias with funnel plots, we will use the trim-and-fill method [37]. Third, we will examine the Hedges’ *d* values of individual effect sizes in a normal-quantile plot. If the effect sizes are from a normal distribution, the data point (effect size of individual studies) will rest near the diagonal of X Y; deviation will suggest publication bias [38].

### Moderator analysis

We will perform moderator analyses to examine whether the variance of study-specific treatment effects is systematically associated with aspects of study designs and/or population characteristics. Proposed moderators include the nature and types of summary information provided in the descriptive norms messages (e.g., information about behavior summarized as proportions, total numbers, mean, or median of the reference population), delivery modality (e.g., personalized feedback, public messaging), and population characteristics (e.g., age and gender distributions, if any). This list of moderators will be further refined based on findings from our thematic syntheses. We will perform a power determination prior to the moderator analyses to aid in the interpretation of the results [39].

## Discussion

This systematic review and the related thematic and meta-analyses will contribute evidence that may enhance understanding regarding the effect of descriptive norms interventions on subgroups with different behavioral patterns prior to intervention exposure. Findings from this study can provide information to practitioners and policy makers who plan to leverage descriptive norms messaging to encourage the adoption and maintenance of improved health and environmental sustainability behaviors. While descriptive norms messaging is common in these applied settings, there is a lack of systematically derived advice on when practitioners or researchers who are designing these interventions should account for possible boomerang effects, and how they can do so most effectively. In particular, this study will provide evidence regarding specific intervention messaging characteristics that are effective and the factors that need to be taken into consideration when designing, planning, and executing such interventions. It is worth emphasizing here that this study will synthesize and integrate empirical evidence across study domains and will provide insights on multidisciplinary collaborations. Complex and interconnected problems such as those arising in the environmental and public health arenas will likely require the examination of rigorous evidence emerging across disciplines to identify effective, interdisciplinary solutions.

Potential limitations of this review include (1) the consideration of studies published only in the English language for inclusion, (2) the restriction of literature searches to the selected databases, (3) the exclusion of studies that did not report the pre-intervention behavior measures. Results will be disseminated through publication in a peer-reviewed journal. Any amendments made to this protocol during the review will be outlined in PROSPERO and reported in the final manuscript.

## Supplementary Information

**Additional file 1:.** PRISMA-P checklist.

**Additional file 2:.** Search strategy details.

**Additional file 3:.** Data to be extracted.

## Data Availability

Not applicable

## Abbreviations

PRISMA-P: Preferred Reporting Items for Systematic Review and Meta-analysis Protocols
PROSPERO: International Prospective Register of Systematic Reviews
RCTs: Randomized controlled trials
RoB2: Revised Cochrane Risk of Bias assessment tool
ROBINS-I: Risk Of Bias in Non-randomized Studies—of Intervention
LQAT: Liverpool Quality Assessment Tool
