# Supplementary material for: Do descriptive norms messaging interventions backfire? Protocol for a systematic review of the boomerang effect
Source: Syst Rev. 2020 Nov 24;9:267. doi: 10.1186/s13643-020-01533-0 (PMC7687726; doi:10.1186/s13643-020-01533-0)
Supplement: Supplementary file 2 — Additional file 2:. Search strategy details. [file 13643_2020_1533_MOESM2_ESM.docx]

**Additional file 2: Search strategy details.**

| Search set | MEDLINE |
| --- | --- |
| 1 | Descriptive norms* ti, ab |
| 2 | “Social Norms”[Mesh] ti, ab |
| 3 | “Social Conformity”[Mesh] ti, ab |
| 4 | “Social Perception”[Mesh]ti, ab |
| 5 | 1-4/OR |
| 6 | “Health Behavior” [Mesh] OR ”Health Knowledge, Attitudes, Practice”[Mesh] OR “Health Education”[Mesh] |
| 7 | “Social Marketing”[Mesh] OR “Health Communication” [Mesh] OR “Health Promotion” [Mesh] OR “Health Promotion” [Mesh] |
| 8 | Environment* ti, ab |
| 9 | 6-8/OR |
| 10 | Limit 9 to Humans |

This is the preliminary search strategy for MEDLINE. We will adapt it for other electronic databases. We will report all search strategies
