# Supplementary material for: Do descriptive norms messaging interventions backfire? Protocol for a systematic review of the boomerang effect
Source: Syst Rev. 2020 Nov 24;9:267. doi: 10.1186/s13643-020-01533-0 (PMC7687726; doi:10.1186/s13643-020-01533-0)
Supplement: Supplementary file 3 — Additional file 3:. Data to be extracted. [file 13643_2020_1533_MOESM3_ESM.docx]

**Additional file 3: Data to be extracted**

| **Field** | **Description** |
| --- | --- |
| **STUDY INFO** |  |
| Person extracting the data |  |
| Date of extraction |  |
| Study ID | Internal unique identifier for each study; assigned after title and abstract screening |
| URL | Link to the online study |
| Primary author of study, year published | Author(year) |
| **STUDY ELIGIBILITY** |  |
| Type of study | Randomized controlled trials; non-randomized controlled trials; controlled before-and-after studies; interrupted-time-series studies; uncontrolled before-and-after studies; and case series (uncontrolled longitudinal studies) |
| Type of intervention | Indicate whether a descriptive norms intervention was used |
| Type of comparison | Descriptive norms intervention vs. no treatment (treatment vs. control); Descriptive norms intervention vs. alternative intervention (treatment vs. alternative counterfactual); Descriptive norms intervention only (pre- vs. post assessments of exposed groups, or interrupted time-series studies or case series studies) |
| Type of outcome measure: Pre- and post-intervention assessments | Indicate whether authors provide both pre and post treatment measures of behaviors |
| **STUDY CHARACTERISTICS** |  |
| Country/Region | Country or countries and region or regions in which the study was conducted |
| Study setting | Setting in which the study was carried out (e.g., in a lab or naturalistic field setting, such as community, school, and healthcare facility) |
| Primary and secondary study outcomes | Description of the primary and secondary outcomes targeted by the study |
| Behavior(s) targeted by the study | Specific behavior(s) targeted by the study and desired direction of the targeted behavior. |
| Domain of targeted behavior(s) | Behavioral domain targeted by the intervention (e.g., health behavior, energy consumption) – to be informed by the thematic synthesis |
| **PARTICIPANT CHARACTERISTICS** |  |
| Target population | Description of targeted population (e.g., college students, resident in a neighborhood, consumer of supermarket) |
| Nationality or nationalities/ethnicities | Nationality or nationalities and ethnicities of the study participants |
| Age | The mean and variance of participants |
| Gender | Gender of participants (single or mixed sex) |
| Educational attainment and/or literacy | Indicate educational attainment, literacy rate, or level of literacy |
| **STUDY DESIGN** |  |
| Type of study design | Classification of study design (e.g., ex-ante randomized controlled or cluster randomized trials, nonrandomized controlled trials, controlled before-and-after studies,  interrupted-time-series studies, uncontrolled before-and-after studies, and case series (uncontrolled  longitudinal studies) |
| Description of study design | Brief description of study design |
| Sample size | Total sample size at pre- and post-treatment, by study arm (as appropriate) |
| Sampling strategy | Description of the strategy used to draw the sample from the target population |
| Duration of study | Number of months from the first pre-intervention data collection moment to the last data collection moment included in the study |
| Season(s) in which data were collected | Local season in which data were collected |
| Duration of exposure | Total time of exposure to the intervention |
| Follow-up interval | Time interval between follow-up visits (e.g., weekly, monthly, six-monthly, annually) |
| Total follow-up duration | Total time between intervention exposure and the final post-exposure data collection moment |
| Reasons for missing data | Reasons for loss of follow up or other missing data |
| Analytical approach to missing data | Describe how missing data were handled analytically |
| Unit of randomization | Unit of randomization and whether the analysis adjusted for clustering if cluster design |
| Outcome variable | The outcome upon which the effect size was calculated |
| Outcome measurement – Measurement modality | Manner in which the outcome behavior was measured (e.g., reported, observed, instrument-recorded) |
| Outcome measurement – Type of outcome (prevalence, degree, or frequency) | Indicate whether the outcome measurement was a binary measure (presence vs. absence of behavior), ordinal measure (always, sometimes, never), count/frequency, or other (specify) |
| **INTERVENTION CHARACTERISTICS** |  |
| General intervention description | Summary of intervention design |
| Descriptive norms messaging | Indicate whether descriptive norms messaging was used as an intervention technique |
| Methods through which descriptive norms messages were disseminated | Indicate the means through which descriptive norms messages were transmitted (e.g., via audio visual [commercial], via audio only [radio], visually via words [advertisement], visually via symbols or other signals [flags, decals, icons]) |
| Message content | The wording of intervention message |
| Descriptive noms messaging framing – qualitative vs. quantitative | Indicate whether descriptive norms messages employed qualitative messaging (majority) vs. quantitative messaging (8 out of 10, 80%) |
| Descriptive norms framing - level of abstraction | Level of abstraction when describing what other people do (e.g., 8 out of 10 vs. 80%) |
| Descriptive norms framing - prevention or promotion | Indicate whether the descriptive information was intended to prevent undesirable/unimproved behavior(s) or promote desirable/improved behavior(s) |
| Duration of intervention exposure | The total amount of time the participants were exposed to the descriptive norms messaging intervention |
| Delivery agent | The agent through which an intervention was delivered (e.g., health worker, government official, celebrity, peer). |
| **STATISTICAL INFORMATION** |  |
| Sample size-counterfactual | Number of people not exposed to the intervention (e.g., in counterfactual arm(s)), as appropriate |
| Sample size-intervention | Number of people exposed to the intervention |
| Effect size measure |  |
| Effect size-effect size (Standard Error) | Cohen's d, which is the estimated effect size of standard mean difference between intervention and counterfactual(s). Standard Error in parenthesis. |
| Effect size-sample of interest | Cohen's d, which is the standard mean difference before and after the intervention amongst those who perform above desired threshold |
| Mean / variance / % engagement of counterfactual(s) [pre/post] | Mean / percentage engaged in desired behavior of counterfactual arm(s), as appropriate |
| Mean / variance / % engagement of intervention [pre/post] | The mean/percentage engaged in desired behavior of intervention groups or group of interest if not RCT |
| Boomerang effect estimate, CI, and p-value | If the study examined the boomerang effect, extract the effect size, CI and p-value |
| Contact author for more information (yes/no) | If yes, extract corresponding author’s email |
